# The Long-Term Association between Physical Activity and Weight Regain, Metabolic Risk Factors, Quality of Life and Sleep after Bariatric Surgery

**DOI:** 10.3390/ijerph19148328

**Published:** 2022-07-07

**Authors:** Cláudia Mendes, Manuel Carvalho, Leandro Oliveira, António Palmeira, Luís Monteiro Rodrigues, João Gregório

**Affiliations:** 1 CRI.COM—Integrated Responsibility Centre for Bariatric Surgery and Metabolic Diseases, Hospital Espírito Santo de Évora, EPE, 7000-811 Evora, Portugal; cmendes@hevora.min-saude.pt (C.M.); mcarvalho@hevora.min-saude.pt (M.C.); 2 CBIOS—Research Center for Biosciences & Health Technologies, Universidade Lusófona, 1749-024 Lisboa, Portugal; leandro.oliveira@ulusofona.pt (L.O.); monteiro.rodrigues@ulusofona.pt (L.M.R.); 3 CIDEFES—Research Center in Sport, Physical Education, Exercise and Health, Universidade Lusófona, 1749-024 Lisboa, Portugal; antonio.palmeira@ulusofona.pt

**Keywords:** physical activity, bariatric surgery, weight regain, quality of life, metabolic risk factors, sleep quality

## Abstract

Bariatric surgery is currently regarded as a safe and effective long-term procedure for the treatment of obesity and related comorbidities. We analyzed the association between physical activity (PA), weight regain, metabolic risk factors and quality of life in patients submitted to bariatric surgery. This study also aimed to preliminarily assess how physical activity and weight regain may be associated with sleep quality and sedentary behavior. This was an observational study, with retrospective data collection and a cross-sectional survey. Retrospective clinical data were collected from a sample of 84 individuals who had undergone bariatric gastric bypass surgery at least five years prior to the study period in an Integrated Responsibility Center for Obesity and Metabolic Diseases Surgery. The survey, developed from validated questionnaires and applied in telephone interviews, focused on health data, associated comorbidities, quality of life, physical activity, sedentary behavior and sleep. Descriptive and comparative statistics were performed with a 95% confidence level. Bariatric surgery induced a significant weight loss in the first year after surgery. Our analysis also revealed that lower levels of PA were associated with weight regain. Quality of life as well as sleep quality were inversely related to weight regain, as well as sedentary behavior in general. Primary and secondary outcomes of bariatric surgery can be better achieved if the practice of PA could be maintained for consecutive years.

## 1. Introduction

Obesity is a widespread public health problem with growing chronicity. It results from numerous concurrent factors, leading to the accumulation of body fat [1]. It is difficult to control and globally compromises health, as it is an important risk factor for multiple diseases [2].

Currently, bariatric surgery is regarded as a safe and effective long-term procedure for the treatment of obesity and its comorbidities [3]. It is the first-choice treatment in morbidly obese patients, even in the presence of other pathologies [4]. Bariatric surgery promotes significant weight loss and the reduction in related comorbidities. In fact, bariatric surgery was key in observing that diabetes remission could be independent of weight loss in patients with type 2 diabetes mellitus (T2DM), even before any significant weight loss [5]. Most importantly, this improvement in the individual’s health is associated with increased quality of life, defined as the individual’s perception and satisfaction with their daily life [6,7,8], and is the most common reason for choosing bariatric surgery. Moreover, it also impacts the subjective quality of sleep and the daytime sleepiness that often persists after surgery [9]. Finally, recent evidence on the association of bariatric surgery and lower risk of all-cause mortality among patients with T2DM has also emerged [10].

The weight reduction after bariatric surgery might last up to two years [11,12]. Weight regain starts at different rhythms and seems to be conditioned by behavioral influences [11]. Poor diet quality and nutritional monitoring and high levels of sedentary behavior are recognized determinants for weight regain [13]. Metabolic risk factors seem to be kept low during the process, although physical activity and exercise will help a better control in the long term [14]. A recent study suggested that physical activity after bariatric surgery might be associated with additional weight loss and more effective long-term weight control [15]. Therefore, it seems clear that an active lifestyle is essential to optimize and maintain weight control after bariatric surgery. However, experimental evidence on the effects of physical activity and monitored exercise on obesity-related outcomes in this specific population is still lacking [14]. Specifically, what constitutes an active lifestyle that is sufficient to prevent weight regain in the population of bariatric surgery patients remains to be clearly understood [12]. There is evidence that an active lifestyle benefits blood glucose levels and insulin responses after a meal [16], but evidence of the impact of bariatric surgery on the functional performance (i.e., the physical capacity to perform everyday tasks, such as climbing stairs, buy groceries or tie a shoelace [17]) of patients remains insufficient.

As previously stated, weight regain is dependent on behavioral influences whose impact becomes more obvious many years after bariatric surgery [18]. However, only a few studies studied weight-regain-associated factors five years after the intervention [19]. Therefore, the main aim of this study was to analyze the association between physical activity and weight regain, metabolic risk factors and quality of life, five years after bariatric surgery. Additionally, we also explored the impact of sedentary behavior on weight regain and the potential relationship among physical activity, weight regain and sleep quality.

## 2. Materials and Methods

### 2.1. Study Design

This is an observational study with two components: a retrospective and a cross-sectional study. As inclusion criteria, participants were required to be over 18 years old and submitted to laparoscopic gastric bypass surgery. Participants were not included if they had post-surgical complications or if they presented any contraindication for the practice of exercise. Patients with psychiatric or neurological disorders and pregnant patients were also not included.

**Figure 1:**
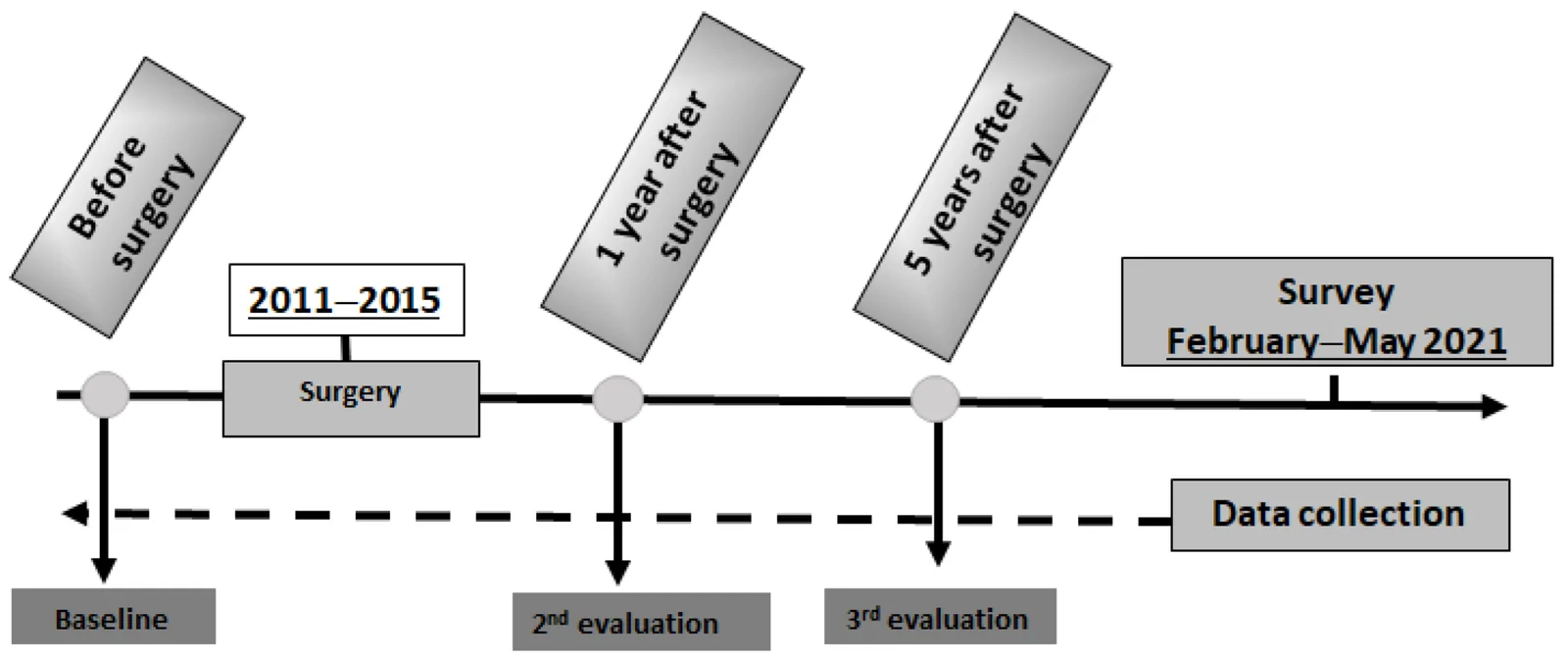
Study design and retrospective data collection points.

Retrospective data from patients who underwent surgery in the Hospital Espírito Santo, Évora, Portugal (HESE, EPE), were collected from electronic health records (EHR), following previous informed consent. Data collection in the EHR focused on before surgery (baseline), one year after surgery (second evaluation) and five years after surgery (third evaluation) (Figure 1). Patients were included if they had surgery between 2011 and 2015. For the cross-sectional study, a survey was performed through telephone interviews conducted between February and May 2021.

### 2.2. Sample

The hospital database included 406 registered patients who had undergone bariatric surgery, specifically, laparoscopic gastric bypass surgery, since 2011. The first step of this surgery is to make a smaller stomach, and the second step is the bypass, where the new stomach is connected to a small part of the intestine (jejunum) [20]. It is a food-restrictive and malabsorptive surgery.

**Figure 2:**
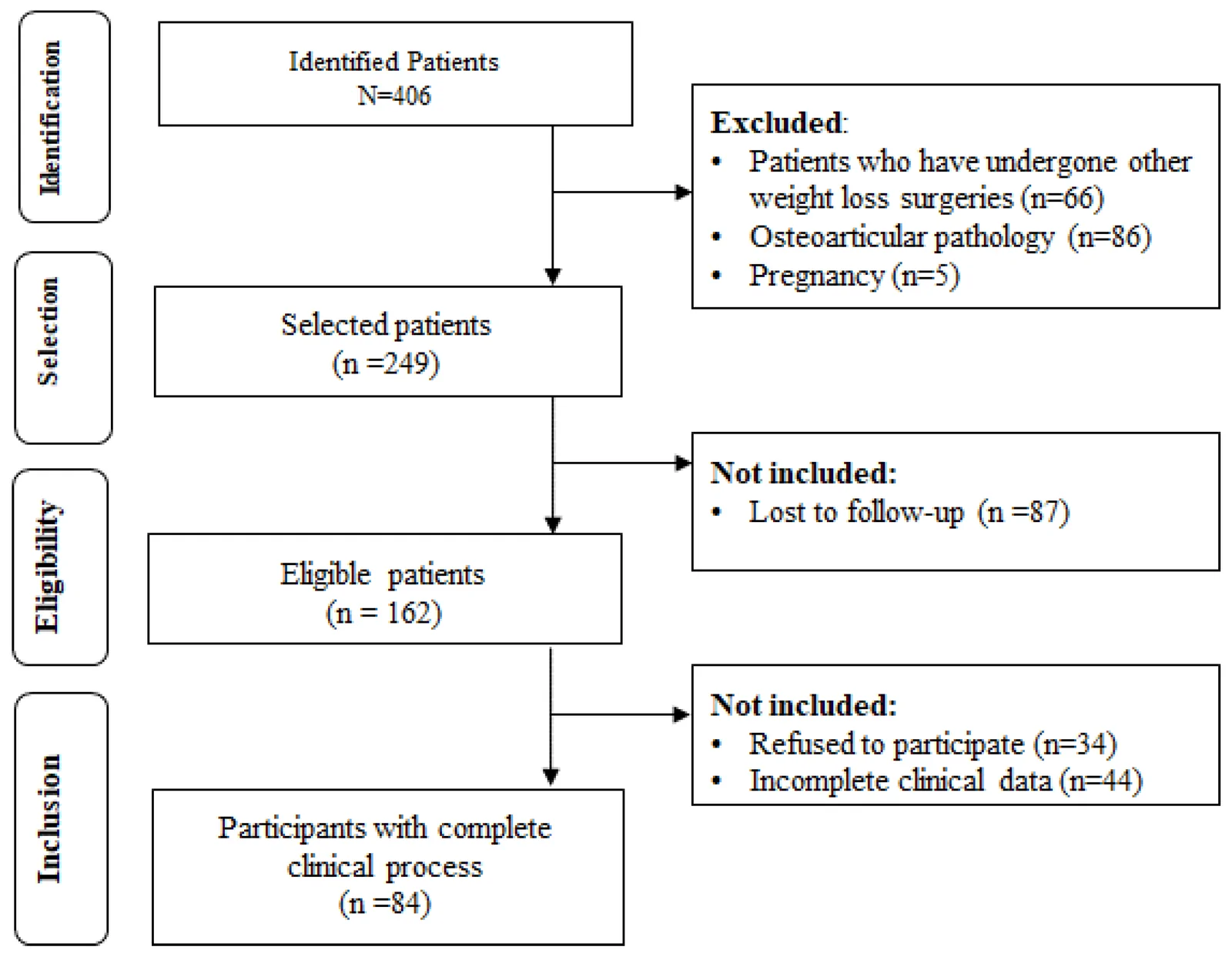
Patient selection process diagram.

After application of the inclusion/non-inclusion criteria, 249 patients were selected. Within this group, no follow-up (post-surgery) data were collected for 87 patients, forcing their exclusion from the study. Thus, 162 patients were eligible (Figure 2). All these patients were contacted, and 128 patients agreed to participate in the study, while 34 refused or were not available to respond. Despite the number of patients who agreed to participate, some of the EHRs did not contain complete clinical or health data corresponding to the evaluation periods of our study. Therefore, these patients were also not considered. Thus, the final sample involved the participation of 84 individuals.

### 2.3. Measures/Instruments

An assessment instrument was developed, which included various questionnaires to collect clinical data, anthropometric parameters and surgical data.

To assess quality of life, the questionnaire “The Impact of Weight on Quality of Life-Lite (IWQOL-Lite)” was used. It is as a self-reported measure, with 31 items, validated for the Portuguese language, specific to obesity. Scores range from 0 to 100, with 100 representing the best quality of life [21].

The Pittsburgh Sleep Quality Index (PSQI) is a sleep quality self-assessment questionnaire. It is a self-rated questionnaire, which assesses sleep quality and disturbances over a 1-month time interval. Nineteen items generate seven “component” scores: subjective sleep quality, sleep latency, sleep duration, sleep efficiency, sleep disturbances, use of sleeping medication and daytime dysfunction. The sum of scores for these components yields one global score [9]. Afterward, it is possible to categorize this variable as either Good (PSQI < 5) or Poor (PSQI ≥ 5) sleep quality [22].

Physical activity level and type of physical activity were assessed by the International Physical Activity Questionnaire (IPAQ). The IPAQ was developed to measure health-related physical activity. The short version, with four levels of physical activity, has been tested extensively and is now used in many international studies [23].

Weight regain (% weight regain) was calculated based on the formula:

$$\frac{\mathrm{current}\;\mathrm{weight}-\mathrm{minimum}\;\mathrm{postoperative}\;\mathrm{weight}}{\mathrm{presurgery}\;\mathrm{weight}-\mathrm{minimum}\;\mathrm{postoperative}\;\mathrm{weight}}\times 100$$

For the purpose of this work, we considered the weight one year after surgery obtained in the EHR as the minimum postoperative weight. There is a considerable ongoing debate around this concept, namely, to establish the adequate threshold to predict surgery failure [24,25,26,27,28,29,30]. In this work, we decided to opt for a conservative approach, namely that 5% of weight regain calculated by this formula was a sign of a significant weight regain. Another way to determine surgery success is through the Body Mass Index (BMI), with BMI < 30 kg/m^2^ being considered an excellent result, between 30 and 35 kg/m^2^, a good result, and >35 kg/m^2^, a failure in the technique or a failure of surgery [31].

### 2.4. Statistical Analysis

Descriptive and comparative statistics were performed with Jamovi software Version 2.2.5 (jamovi project, Sydney, Australia). A 95% level of confidence was adopted throughout the analysis. Normality was analyzed with the Shapiro–Wilk test and based on this result, the most appropriate statistical tests were selected. Chi-square or Fisher’s exact tests for categorical variables, parametric (*t*-test and repeated-measures ANOVA) or non-parametric tests (Mann–Whitney) for continuous variables, were used where appropriate.

## 3. Results

The final sample used for this study included 84 participants who underwent surgery between 2011 and 2015 with the gastric bypass surgical technique. In this sample, there was a large majority of women (n = 77; 91.7%). The mean age (±SD) of the sample was 50.1 y.o. (±8.8). More than a third of the sample had hypertension, and a quarter were diabetic patients before surgery (Table 1).

Women were significantly younger than men (49.5 ± 8.5 vs. 56.9 ± 8.9; *p*-value = 0.032). However, relative to the other risk factors at baseline, only the initial weight was different between men and women.

Looking into surgery success if using the BMI attained one year and five years after surgery, 77.38% of patients achieved an excellent result (<30 kg/m^2^) at one year, but only 48.81% kept that result at the five-year mark. As for the weight regain, we found that 53.57% of patients had more than 5% of weight regain five years after surgery.

Regarding the evolution of risk factors in the five years post-surgery (Table 2), it is clear that one year after surgery, there was an improvement in all risk factors. However, this evolution is clearly different in each of the weight regain groups. In the case of cholesterol and glycaemia, only people who had more than 5% of weight regain achieved a significant improvement 1 year after surgery. After five years, these two risk factors present a rebound that was sufficient to return to baseline values. In the case of mean blood pressure, both groups of patients managed to sustain the improvement after five years.

**Figure 3:**
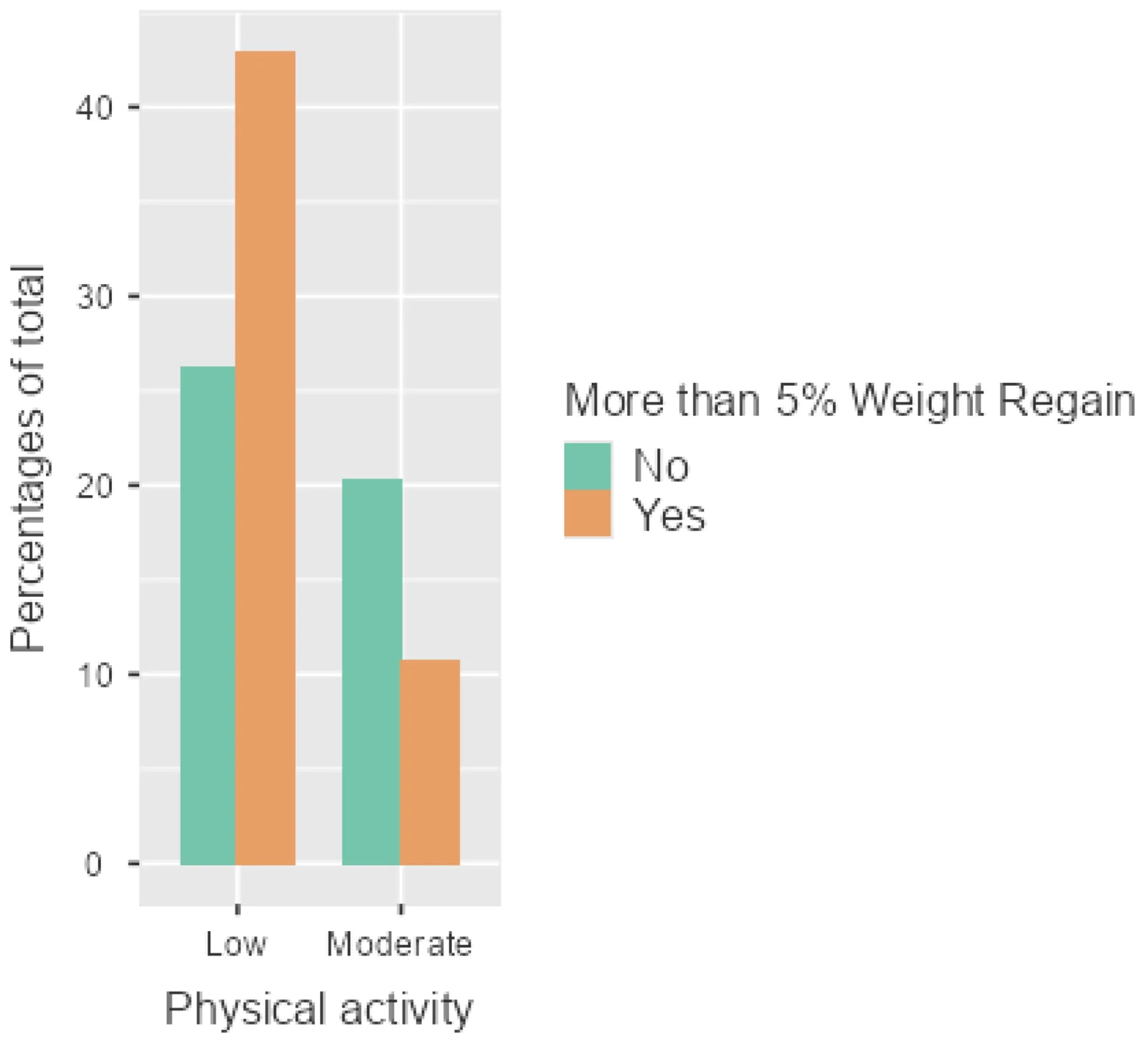
Association between weight regain (n = 45) and low physical activity (n = 58). Unadjusted odds ratio 3.09 (IC 95%: 1.18–8.12; *p* = 0.020)).

Physical activity was characterized with three different levels—low, moderate or high physical activity—according to the description of the IPAQ questionnaire. In this sample, only two levels were present—low physical activity (69.9% of the sample) and moderate physical activity (30.9%). A statistically significant association of low physical activity with more than 5% of weight regain was found (χ^2^ = 5.44, *p* = 0.020) (Figure 3). Adjusting for age, sex and initial weight, the odds ratio for this association was 3.25 (IC 95%: 1.20–8.83; *p* = 0.021)).

The evaluation of quality of life included data on self-esteem, physical function, quality of work activity, sexual life and public behaviors. Moderately active participants had significantly higher values (i.e., better scores) in all dimensions of quality of life (Table 3). Additionally, participants who did not have weight regain had higher values in all dimensions of quality of life, except in the “Work” dimension.

The results of sleep quality showed that many of the patients reported having sleep difficulties (38%). The reasons given for these difficulties were work, pain and family concerns. However, most of the patients had no difficulty staying awake during meals or while driving (82%). Overall, more than half (53.6%) of the patients had a quality of sleep considered poor (PSQI ≥ 5) [22]. When testing the association of sleep quality with other independent variables (Table 4), both the practice of physical activity and a body mass index below 30 kg/m^2^ showed a significant association with good quality of sleep. In the multivariate logistical model adjusted for sex, age and physical activity, patients who achieved and maintained a BMI below 30 kg/m^2^ five years after bariatric surgery were 2.8 times more likely to present good quality of sleep (IC 95%: 1.10–7.27; *p* = 0.031)).

## 4. Discussion

The main objective of the present study was to analyze the association between the regular practice of physical activity and weight regain in patients who had undergone bariatric surgery, since, in this context, weight regain is one of the most important indicators of surgical failure. Associations with sedentary behavior, metabolic risk factors, quality of life and sleep quality were also evaluated.

The results of this research showed that a higher level of physical activity was associated with lower weight regain. This effect was also noticed in the dimensions of quality of life, which were consistently higher in participants who were active and kept their weight regain below 5% after surgery (Table 3). Maintaining the BMI below 30 kg/m^2^ was associated with better sleep quality (Table 4). Metabolic risk factors’ evolution was similar across all patients who underwent bariatric surgery (Table 2). However, there were significant differences in the evolution of glycaemia, cholesterol and mean blood pressure across the weight regain groups. Only the mean blood pressure improvement was maintained in both weight regain groups five years after the surgery. 

Metabolic risk factors, when present, infer important repercussions on comorbidities, namely diabetes, hypertension and dyslipidemia [32]. The improvement in the metabolic risk factors following surgery seen in the present study adds to the evidence sustaining bariatric surgery as an effective treatment for comorbidities of obese patients, regardless of weight regain and physical activity [33,34]. It is interesting to notice that patients that showed more than 5% of weight regain also had a significant improvement of glycaemia and cholesterol one year after surgery. It is known that the positive evolution of metabolic risk factors with combined surgical procedures might be related to restriction and deficient nutrient absorption [35]. It is, therefore, possible that the observed rebound might be due to insufficient support in attaining new eating habits that are often too different from the previous habits. Patients also seem to expect that bariatric surgery will help them sustain a long-term control of their eating habits and weight, but as soon as the weight regain starts, the confidence and improvements in eating behaviors are replaced by a sense of loss of control [27]. Patients in these conditions describe increased hunger and decreased satiety, a possible indication that hormonal and metabolic changes occur in the long term after surgery.

Patients’ reports of low or moderate physical activity are in line with what has been reported in several studies stating that bariatric surgery patients fall short of The European Association for the Study of Obesity (EASO) recommendations regarding the practice of physical activity for the prevention of weight regain in the postoperative period [36]. Moderately active patients were more successful in keeping their weight regain controlled at five years after bariatric surgery, which is in line with what has been reported by other authors [14]. Again, none of these patients benefited from a structured physical exercise program. Experience shows that the practice of physical activity in the post-surgery period in these patients is initiated autonomously, without professional support or monitoring. The association of more weight regain with less reported physical activity suggests the need for a professional follow-up of these patients by a physical activity therapist [37].

Quality of life is, in most cases, the primary reason why patients seek help and decide to undergo this type of surgery to treat obesity [38]. Our results show that all the dimensions of quality of life are inversely related to weight regain and directly associated with physical activity (Table 3). This aligns with previously published work reporting improvements in the quality of life of bariatric surgery patients that involved social life and work related with the practice of physical activity [39]. The sleep quality questionnaire indicated that the majority of these patients have poor sleep quality. Sleep quality was associated with physical activity and keeping the BMI below 30 (Table 4). This is also in line with the literature, which shows that as early as 6 months postoperatively, there is an improvement in sleep and (lower) depression levels also associated with physical activity [40].

Another important aspect in the analysis of these results is to contribute to the discussion about reviewing the definition of relevant outcomes that determine the success of the bariatric surgery intervention. Here, we considered a weight regain greater than 5% of the total weight loss as the outcome of interest, in line with previous publications [25,27,41,42]. Evidence shows that weight regain occurs, on average, 27 months after bariatric surgery and is more pronounced in patients with low levels of physical activity [40]. However, professionals’ and patients’ expectations regarding bariatric surgery outcomes are not necessarily the same [43,44,45]. Professionals are more concerned with weight and comorbidities, while patients focus on quality of life and well-being in general, with feelings of demotivation linked to unrealistic prospects of weight loss after surgery [46]. To align with patients’ expectations, a movement toward patient-reported outcomes measures (PROM) to assess the success of this intervention is suggested. PROM are directly reported by patients and focus on their feelings and functioning related to their health condition or therapy [44,47,48]. They are distinct from clinical outcomes and most of the time include the assessment of several dimensions of quality of life or satisfaction [49]. Using PROM would allow tailoring interventions made during the follow-up of these patients, potentially improving their health outcomes. This study assessed some PROM to evaluate their relationship with physical activity and weight regain. Future studies should focus on identifying bariatric surgery PROM that may predict surgery failure to add to the body of evidence on the subject. 

Some limitations of this study should be recognized: (a) the number of participants is small regarding the global hospital sample. However, our 20% recruitment rate with complete data can be seen as an adequate sample to provide information on patients from this particular center; (b) the use of self-reported retrospective data obtained by telephone interviews may be prone to memory bias; (c) the barriers and facilitators of physical activity were not fully identified; (d) data about diet-related variables were not collected, hampering the possibility to assess the influence of diet on weight regain or on the practice of physical activity; and (e) due to the cross-sectional nature of this study, one can only claim that the association between PA and lower weight regain exists. For the remaining outcomes (metabolic risk factors and quality of life dimensions), the confounding effect of this association limits our conclusions. Nevertheless, the benefits of PA are widely consensual; recent trials seem to confirm the beneficial potential of regular physical exercise, including walking, in prevention and recovery from different disease conditions and in the control of several metabolic risk factors [50,51,52].

To overcome these limitations, future studies should be based on a prospective design, allowing for a better determination of the impact of diet and physical activity on the success of post-surgery weight maintenance, improvement of metabolic risk factors and quality of life.

## 5. Conclusions

Low physical activity is associated with weight regain after bariatric surgery. In the long term, maintaining a moderate physical activity practice will predict lower weight regain. Metabolic risk factors improve after surgery, although only the improvement in mean blood pressure is maintained in the long term. Lower weight regain is associated with better quality of life and better sleep quality. These results reinforce the importance of an active lifestyle promotion integrated intervention as part of the approach to obesity treatment after bariatric surgery. 

## Figures and Tables

**Table 1:** Baseline (pre-surgery) characteristics of the sample.

	All Subjects (n = 84)
Sex (Women%)	91.67%
Age (years) *	50.11 (8.76)
Marital Status	
Single	15.48%
Married	59.52%
Divorced	22.62%
Widow	2.38%
Initial weight (kg) *	113.86 (17.76)
Initial BMI (kg/m^2^) *	44.79 (4.99)
Cholesterol (mg/dL) *	167.51 (39.90)
Glycaemia (mg/dL) *	95.93 (25.70)
MBP (mm Hg) *	96.24 (13.40)
Hypertension prevalence	36.90%
Diabetes prevalence	25.00%
Dyslipidemia prevalence	35.71%
Sleep apnea prevalence	9.52%

BMI: Body mass index. * Mean (SD).

**Table 2:** Comparison of the mean (±SD) evolution of metabolic risk factors before and after surgery according to percentage of weight regain. Repeated-measures ANOVA with the Tukey or Scheffe tests for pairwise comparisons.

Risk Factors	Weight Regain <5% (n = 39) >5% (n = 45)	Baseline	Year 1	Baseline vs. Year 1 *p*-Value	Year 5	Baseline vs. Year 5 *p*-Value	Year 1 vs. Year 5 *p*-Value
**weight (kg)**	<5%	113.2 ± 19.7	74.7 ± 9.3	**<0.001**	72.5 ± 9.9	**<0.001**	0.255
	>5%	114.4 ± 16.1	75.1 ± 11.6	**<0.001**	83.6 ± 13.1	**<0.001**	**<0.001**
	*p*-value	1.000	1.000		**<0.001**		
**BMI (kg/m^2^)**	<5%	44.3 ± 5.1	27.1 ± 3.0	**<0.001**	28.2 ± 3.2	**<0.001**	0.106
	>5%	45.2 ± 4.9	27.9 ± 4.4	**<0.001**	32.8 ± 4.8	**<0.001**	**<0.001**
	*p*-value	0.984	0.955		**<0.001**		
**cholesterol (mg/dL)**	<5%	159.3 ± 39.5	155.9 ± 37.5	0.985	161.3 ± 38.7	0.999	0.826
	>5%	174.6 ± 39.3	160.8 ± 31.2	**0.048**	174.4 ± 34.5	1.000	**0.016**
	*p*-value	0.486	0.986		0.573		
**glycaemia (mg/dL)**	<5%	95.4 ± 25.0	87.9 ± 12.0	0.244	94.8 ± 22.0	1.000	0.038
	>5%	96.4 ± 26.6	84.9 ± 8.2	**0.007**	93.6 ± 19.0	0.885	**0.001**
	*p*-value	1.000	0.752		1.000		
**MBP (mm Hg)**	<5%	95.0 ± 14.2	87.2 ± 10.1	**0.001**	84.6 ± 9.5	**<0.001**	0.483
	>5%	97.3 ± 12.7	86.6 ± 7.8	**<0.001**	88.4 ± 10.7	**<0.001**	0.739
	*p*-value	0.972	0.999		0.529		

BMI: Body mass index. MBP: Mean blood pressure. Bold—*p* < 0.05.

**Table 3:** Differences in the dimensions of quality of life between physical activity groups and weight regain groups. Scores are means ± SD. Differences assessed with the Mann–Whitney test.

	Level of Physical Activity			>5% Weight Regain		
	Low (n = 58)	Moderate (26)	*p*	Yes (n = 45)	No (n = 39)	*p*
Score QoL Total	85.7 ± 22.7	98.8 ± 5.3	**0.005**	84.4 ± 24.3	95.9 ± 10.6	**0.014**
Physical Function	84.7 ± 24.9	97.9 ± 10.7	**0.003**	82.4 ± 27.5	96.2 ± 10.3	**0.005**
Self-Esteem	84.9 ± 24.7	99.0 ± 4.9	**0.004**	83.2 ± 26.7	96.3 ± 10.0	**0.017**
Sexual Activity	83.9 ± 26.0	99.0 ± 4.9	**0.003**	83.2 ± 27.9	94.9 ± 12.7	**0.039**
Public Behavior	87.1 ± 21.9	99.2 ± 2.7	**0.009**	86.7 ± 23.1	95.6 ± 11.4	**0.047**
Work	89.9 ± 19.6	100.0 ± 0.0	**0.005**	90.4 ± 2.4	96.0 ± 11.2	0.232

QoL: Quality of Life. Bold—*p* < 0.05.

**Table 4:** Association between sleep quality and sex, physical activity, weight regain and BMI variables.

	PSQI		
	Poor	Good	*p*-Value
All patients (%)	53.6	46.4	
Age (years)	49.2 (9.4)	51.2 (7.9)	0.311
Sex			
Men (%)	42.9	57.1	0.553
Women (%)	54.5	45.6	
More than 5% WR			
No (%)	46.1	53.9	0.204
Yes (%)	60.0	40.0	
Physical Activity			
Low (%)	62.1	37.9	0.020
Moderate (%)	34.6	65.4	
BMI at 5 years			
<30 kg/m^2^ (%)	39.0	61.0	0.009
≥30 kg/m^2^ (%)	67.4	32.6	

PSQI: Pittsburgh Sleep Quality Index. WR: Weight regain. BMI: Body Mass Index.

## Data Availability

The data presented in this study are available on request from the corresponding author. The data are not publicly available due to privacy.
